# Temperature-Induced Changes in Reperfused Stroke: Inflammatory and Thrombolytic Biomarkers

**DOI:** 10.3390/jcm9072108

**Published:** 2020-07-04

**Authors:** Paulo Ávila-Gómez, Pablo Hervella, Andrés Da Silva-Candal, María Pérez-Mato, Manuel Rodríguez-Yáñez, Iria López-Dequidt, José M. Pumar, José Castillo, Tomás Sobrino, Ramón Iglesias-Rey, Francisco Campos

**Affiliations:** 1 Clinical Neurosciences Research Laboratory (LINC), Health Research Institute of Santiago de Compostela (IDIS), E15706 Santiago de Compostela, Spain; paulo.avila.gomez@sergas.es (P.Á.-G.); pablo.hervella.lorenzo@sergas.es (P.H.); andres.alexander.da.silva.candal@sergas.es (A.D.S.-C.); jose.castillo.sanchez@sergas.es (J.C.); tomas.sobrino.moreiras@sergas.es (T.S.); 2 Neuroscience and Cerebrovascular Research Laboratory, Department of Neurology and Stroke Center, La Paz University Hospital, Neuroscience Area of IdiPAZ Health Research Institute, Universidad Autónoma de Madrid, E28046 Madrid, Spain; mery19832005@yahoo.es; 3 Stroke Unit, Department of Neurology, Health Research Institute of Santiago de Compostela, Hospital Clínico Universitario, 15706 Santiago de Compostela, Spain; manyanez@yahoo.es (M.R.-Y.); iriaalejandralopez@googlemail.com (I.L.-D.); 4 Department of Neuroradiology, Hospital Clínico Universitario, Health Research Institute of Santiago de Compostela (IDIS), E15706 Santiago de Compostela, Spain; josemanuel.pumar@usc.es

**Keywords:** biomarkers, ischemic stroke, recanalization therapy, reperfusion, temperature

## Abstract

Although hyperthermia is associated with poor outcomes in ischaemic stroke (IS), some studies indicate that high body temperature may benefit reperfusion therapies. We assessed the association of temperature with effective reperfusion (defined as a reduction of ≥8 points in the National Institute of Health Stroke Scale (NIHSS) within the first 24 h) and poor outcome (modified Rankin Scale (mRS) > 2) in 875 retrospectively-included IS patients. We also studied the influence of temperature on thrombolytic (cellular fibronectin (cFn); matrix metalloproteinase 9 (MMP-9)) and inflammatory biomarkers (tumour necrosis factor-alpha (TNF-α), interleukin 6 (IL-6)) and their relationship with effective reperfusion. Our results showed that a higher temperature at 24 but not 6 h after stroke was associated with failed reperfusion (OR: 0.373, *p* = 0.001), poor outcome (OR: 2.190, *p* = 0.005) and higher IL-6 levels (OR: 0.958, *p* < 0.0001). Temperature at 6 h was associated with higher MMP-9 levels (R = 0.697; *p* < 0.0001) and effective reperfusion, although this last association disappeared after adjusting for confounding factors (OR: 1.178, *p* = 0.166). Our results suggest that body temperature > 37.5 °C at 24 h, but not at 6 h after stroke, is correlated with reperfusion failure, poor clinical outcome, and infarct size. Mild hyperthermia (36.5–37.5 °C) in the first 6 h window might benefit drug reperfusion therapies by promoting clot lysis.

## 1. Introduction

Temperature is a long-known pivotal factor in the development and progression of neurological injuries, particularly in the field of stroke, where approximately 50% of patients develop hyperthermia (or fever) within the first 24 h [1,2]. Indeed, higher temperatures during the acute phase of ischaemic stroke (IS) have been associated with greater infarct volumes and worse functional outcomes at 3 months [2,3,4,5,6,7,8]. Therapeutic hypothermia helps to preserve tissue energy and halts several cell death mechanisms; thus, it is currently considered one of the most promising neuroprotective approaches in preclinical models of stroke [9,10]. Nonetheless, although therapeutic hypothermia has well-established neuroprotective effects in specific conditions, its translatability to the human clinic remains elusive [11,12].

Besides mechanical devices, reperfusion drug therapy with recombinant tissue plasminogen activator (rtPA) remains the treatment of choice during the acute phase of an ischaemic event, although its use is limited due to the risk of haemorrhage [13,14,15]. In this regard, body temperature also has a relevant influence on the efficacy of thrombolytic therapy. Paradoxically, although hyperthermia has been associated with poor outcomes and an increased risk of developing haemorrhagic transformation (HT) after rtPA treatment [2], some studies have shown that high body temperatures could have a beneficial effect on reperfusion therapies by enhancing the thrombolytic activity of rtPA [16,17,18]. For instance, previous studies have reported increased fibrinolysis using streptokinase at higher temperatures [19], while lower clot lysis was observed at lower temperatures when rtPA was added to clot suspensions [20]. In patients with acute lower limb ischaemia treated with catheter-directed thrombolysis, heating the rtPA also resulted in faster clot lysis [21]. Therefore, it is not clear if the improvement in clot lysis and efficacy of reperfusion can overcome the deleterious effect of hyperthermia on stroke outcome in rtPA-treated patients.

In this regard, it has been widely reported that, although reperfusion is associated with improved patient outcomes, it can also exacerbate temperature-dependent processes related to blood–brain barrier disruption and post-stroke inflammatory response, leading to increased brain damage [22]. For instance, rtPA upregulates matrix metalloproteinase 9 (MMP-9) expression [23], which is associated with HT and poor outcome in IS [24], and hyperthermia exacerbates the destruction of microvascular integrity by increasing MMP-9 activity [25]. Our group has also reported that circulating levels of fibronectin (cFn) predicted rtPA-associated HT after systemic thrombolysis [26]. Moreover, we have previously demonstrated that higher levels of proinflammatory markers such as interleukin 6 (IL-6) and tumour necrosis factor-alpha (TNF-α) were associated with poor outcomes and larger infarcts in IS patients with hyperthermia [27] and rtPA treatment [28].

Given the conflicting evidence on the effect of temperature on thrombolytic-reperfusion, we hypothesised that higher body temperature during the acute phase of an IS could have a beneficial effect on reperfusion therapies and patient outcomes. Thus, this work aimed to study the influence of temperature on the recanalisation effectiveness and functional outcome at 3 months in a population of IS patients receiving reperfusion therapies. To further support our analysis, we evaluated the effect of temperature on the levels of fibrinolytic and inflammatory activity biomarkers and their impact on the effectiveness of reperfusion. As a secondary objective, we analysed the influence of temperature on the percentage of patients with HT and infarct volume.

## 2. Materials and Methods

### 2.1. Study Design

This retrospective observational study was conducted on a registry of patients with IS included consecutively and prospectively from our databank. The patients were admitted to the Stroke Unit of the University Clinical Hospital of Santiago de Compostela (Spain) from January 2008 to December 2018. The study was performed in accordance with the principles of the Declaration of Helsinki of the World Medical Association and approved by the Research Ethics Committee of Santiago (project identification code 2019/616). Informed consent was obtained from each patient or their relatives after providing a full explanation of the procedures.

### 2.2. Inclusion Criteria

All patients included in this study met the following criteria: (1) authorisation for the anonymous use of their data for research; (2) magnetic resonance imaging (MRI) or computed tomography (CT) study at inclusion and between days four and seven; (3) hemispheric location; (4) patients undergoing reperfusion treatment with rtPA alone or in combination with thrombectomy; (5) patients with a latency time between stroke onset and inclusion of ≤6 h; and (6) a minimum follow-up (face-to-face or telephone) duration of 3 months. Patients who met any of the following criteria were not considered for the study: (1) institutionalised patients, (2) comorbidity and life expectancy <1 year, (3) without subsequent diagnostic confirmation, (4) lacunar infarctions, and (5) loss to follow-up at 3 months.

### 2.3. Blood Samples and Biomarker Assays

Biochemistry, haematology, and coagulation tests were performed in the central laboratory of the hospital. Measurements were made from blood samples obtained in the first 6 h after admission and the next 24 ± 6 h. Although the biomarker analysis was not performed simultaneously, it was supervised by the same researchers and carried out using the same standardised methods. For this study, we selected MMP-9 and cellular cFn as markers associated with thrombolytic activity [24,26], while IL-6 and TNF-α were selected as markers associated with inflammatory activity [27]. For these molecular assessments, venous blood samples were collected in vacutainer tubes (Becton Dickinson, San Jose, CA, USA) in the first 6 h after symptom onset (always after the administration of the thrombolytic bolus) and at 24 ± 6 h. After allowing the sample to clot for 60 min, the blood samples were centrifuged at 3000× *g* for 10 min and the serum was immediately aliquoted, frozen, and stored at −80 °C until analysis.

Serum levels of IL-6 were measured by enzyme-linked immunosorbent assay (ELISA) (BioLegend, San Diego, CA, USA) with a minimum assay sensitivity of 1.6 pg/mL and an intra- and inter-assay coefficient of variation (CV) of 5.0% and 6.8%, respectively, following the manufacturer’s instructions. TNF-α was measured using an immunodiagnostic IMMULITE 1000 System (Siemens Healthcare Global, Los Angeles, CA, USA) with a minimum assay sensitivity of 1.7 pg/mL, an intra-assay CV of 3.5%, and an inter-assay CV of 6.5%. Finally, serum levels of active MMP-9 (GE Healthcare, Amersham, UK, Little Chalfont, Buckinghamshire, UK) and cFn (BioHit, Helsinki, Finland) were measured using commercial ELISA kits following the manufacturer’s instructions. The intra- and inter-assay CVs were <8%. All biomarkers were evaluated within the first 3 months after blood sample collection.

### 2.4. Clinical Scale, Temperature and Therapeutic Management

All patients were admitted to the stroke unit and were treated under the Spanish Neurological Society protocol [29] by trained neurologists experienced in cerebrovascular diseases. The intensity of the neurological deficit was determined by the National Institute of Health Stroke Scale (NIHSS) upon admission to the Stroke Unit. Neurological improvement, defined as a reduction of ≥ 8 points in the NIHSS in the first 24 h, was used as a clinical marker of effective reperfusion [13]. Functional outcome was assessed at 3 months ± 15 days (face-to-face in 80.8% of the sample) using the modified Rankin Scale (mRS) (mRs categorised as poor outcome >2). Both scales were evaluated by internationally certified neurologists. Temperature was measured by the nursing staff every 6 h. For this analysis, the axillary temperature was measured at the time of admission and at 6 and 24 h. Patients with temperatures ≥37.5 °C were treated with paracetamol (500 mg p.o.) or metamizole (2 g i.v.) every 6 h.

### 2.5. Neuroimaging Studies

Cerebral CT or MRI studies were performed on and between days 4 and 7. All neuroimaging studies were supervised by the same neuroradiologist. Symptomatic HT was assessed at the time of recording the neurological worsening and, in any case, in the follow-up CT. HT was defined as symptomatic when it was associated with early neurological deterioration (worsening of at least 4 points in the NIHSS during the first 48 h after stroke onset). HT was classified according to the European Cooperative Acute Stroke Study (ECASS) III [30] criteria as follows: haemorrhagic infarction type 1 (HI1) was defined as small petechiae along the infarct margins, HI type 2 (HI2) was defined as more confluent petechiae within the infarct area but without space-occupying effect, parenchymal haemorrhage type 1 (PH1) was defined as blood clots not exceeding 30% of the infarct with some mild space-occupying effect, and PH type 2 (PH2) as blood clots exceeding 30% of the infarct area with significant space-occupying effect. The PH1 and PH2 groups were considered to have severe HT. The initial lesion volume was determined upon admission by diffusion-weighted imaging (DWI) through an automatic planimetric method. Lesion volume was determined in the follow-up CT using the ABC/2 method [31] until 2016, and then by automated planimetric method.

### 2.6. Endpoints

The main outcome variables were effective reperfusion (defined as a reduction of ≥8 points in the NIHSS in the first 24 h) and poor patient outcomes (mRS >2). Mild hyperthermia was defined as an axillary temperature between 36.5 °C and 37.5 °C. As secondary endpoints, we also analysed the association between effective reperfusion and serum levels of thrombolytic (MMP-9, cFN) and inflammatory (IL-6, TNF-α) biomarkers. Finally, we also determined the association between infarct volume and HT with temperature.

### 2.7. Statistical Analysis

In the descriptive analysis, categorical variables were expressed as frequencies and percentage and as means (standard deviation (SD)) or median and interquartile range (25th and 75th percentiles) for the continuous variables, depending on their adjustment to a normal distribution. The normality of the sample was determined by Kolmogorov–Smirnov tests with Lilliefors correction. Statistical inference was then performed with chi-square, Student’s t, or Mann–Whitney tests according to the nature of the contrast variable and its adjustment to normality. Bivariate correlations were performed using Pearson’s correlations for normally distributed variables.

The association of temperature (at admission and 6 and 24 h) with effective reperfusion, outcome at 3 months, HT, and serum levels of the studied biomarkers were assessed using logistic regression analysis models and the correlation between temperature and infarct volume was analysed by linear multivariate regression. Each model was adjusted for the independent variables in the bivariate analysis. The optimal cut-off points were calculated in the variables of interest using receiver operative curve (ROC) analysis. The results were expressed as adjusted odds ratios (ORs) or B with their respective 95% confidence intervals (CIs). *p*-Values < 0.05 were considered statistically significant in all tests. All analyses were conducted using IBM SPSS Statistics for Macintosh, version 20.0 (IBM Corp, Armonk, NY, USA).

## 3. Results

### 3.1. Sample Description

The sample is described in Table 1. This study included a total of 875 patients (mean age, 72.0 ± 12.5 years; 45.9% females) with acute IS. The mean temperatures at admission and 6 and 24 were 36.2 ± 0.6 °C, 36.6 ± 0.7 °C, and 36.5 ± 0.7 °C, respectively. Regarding the aetiology of the stroke, 43.5% were of cardioembolic origin, followed by atherothrombotic (23.5%) and lacunar (1.3%). Undetermined origin or other origins were reported in 31.7% of cases. Systemic fibrinolysis with rtPA was the reperfusion treatment of choice in 91.1% of cases, and intravenous or i.a. thrombolysis combined with thrombectomy in the remaining 8.9% of cases.

### 3.2. Analysis of the Association between Temperature and Effective Reperfusion

**Figure 1:**
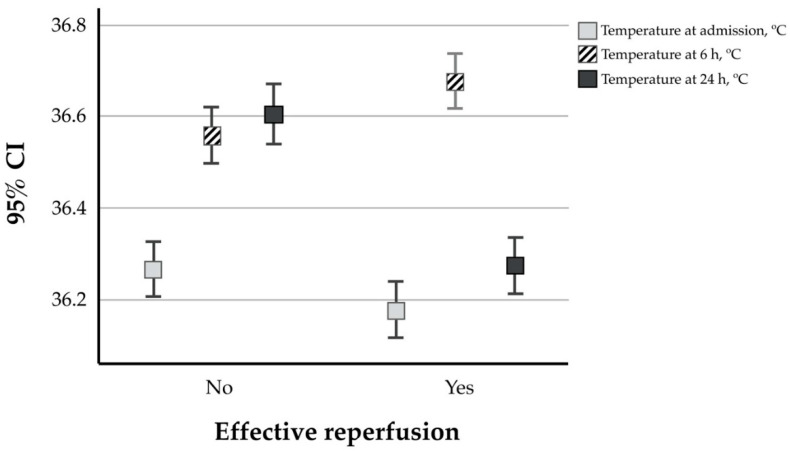
Temperatures at admission and 6 h 24 h in relation to reperfusion effectiveness.

Among the studied population, 44% of the patients had effective reperfusion, while the remaining 56% did not (385 vs. 490 patients). The patients with effective reperfusion showed lower temperatures both at admission and at 24 h. Interestingly, a higher temperature at 6 h was associated with successful reperfusion (Figure 1).

Pearson bivariate correlation analysis performed for the 3 different measurements showed a negative correlation between higher body temperature at admission (*r* = −0.77, *p* = 0.022) and at 24 h (*r* = −0.266; *p* < 0.0001) with effective reperfusion, but no association was observed for the temperature at 6 h (*r* = 0.055; *p* = 0.103). Subsequently, we performed a logistic regression analysis, adjusted for variables that showed significant differences in the bivariate analysis (age, sex, onset-treatment time, blood glucose, atrial fibrillation, NIHSS at admission, leukoaraiosis, and HT) for effective reperfusion. Logistic regression analysis showed a non-significant association between the effectiveness of reperfusion therapy and higher body temperature at 6 h (OR: 1.178; 95% CI: 0.934–1.484; *p* = 0.166). Conversely, higher body temperature at admission and 24 h was negatively associated with effective reperfusion (OR: 0.700; 95% CI: 0.552–0.887; *p* = 0.003; OR: 0.373 and 95% CI: 0.290–0.480; *p* = 0.001) (Table 2).

### 3.3. Evaluation of Temperature and Functional Outcomes at 3 Months

**Figure 2:**
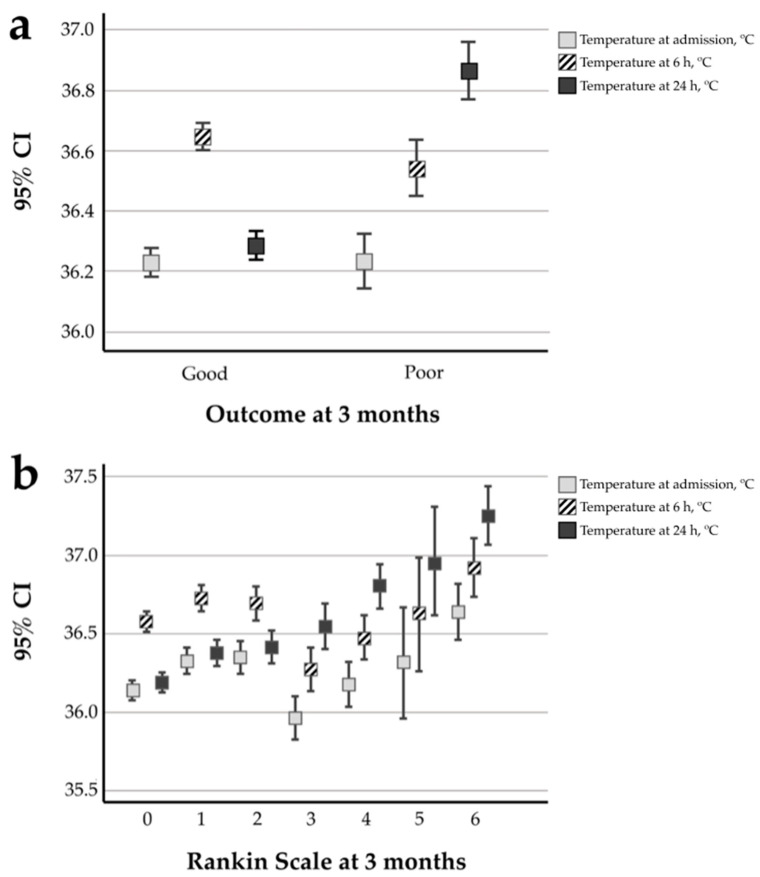
Evaluation of functional outcomes at 3 months. (**a**) Temperatures at admission and 6 and 24 h in relation to patient outcomes. (**b**) Temperatures at admission and 6 and 24 h in relation to modified Rankin Scale scores at 3 months.

Among the 875 patients studied, 606 (69.3%) showed good outcomes at 3 months compared to the remaining 269 (30.7%) patients. Lower temperatures at admission and 24 h were associated with better outcomes at 3 months. Conversely, a higher body temperature at 6 h was associated with good outcomes at 3 months compared to those in the poor outcome group (Figure 2).

Logistic regression models were used to evaluate poor outcome at 3 months in relation to the temperatures at the three timepoints and adjusted by the variables that showed clinical and statistical significance in the model. The analysis showed no association with basal body temperature (OR 0.817, 95% CI 0.475–1.405, *p* = 0.948) and temperature at 6 h (OR 0.792, 95% CI 0.465–1.350, *p* = 0.792). However, the temperature at 24 h was significantly associated with poor patient outcomes at 3 months (OR 2.190, 95% CI 1.264–3.793, *p* = 0.005) (Table 3).

### 3.4. Determination of Temperature-Induced Biomarkers of Thrombolytic and Inflammatory Activity and Their Associations with Effective Reperfusion

cFN and MMP-9 serum levels showed a stronger correlation with temperature at 6 h (*r* = 0.701; *p* < 0.0001; *r* = 0.697; *p* < 0.0001) compared to IL-6 and TNF-α (*r* = 0.113; *p* = 0.001; *r* = 0.223; *p* < 0.0001). Similarly, a greater association was observed between IL-6 and TNF-α levels and temperature at 24 h (*r* = 0.712; *p* < 0.0001; *r* = 0.602; *p* < 0.0001) compared to those for cFN and MMP-9 (*r* = 0.195; *p* < 0.0001; *r* = 0.342; *p* < 0.0001). Based on these findings, we performed multivariate analysis for each biomarker according to their most prominent correlation in relation to effective reperfusion. The analysis showed that MMP-9 levels at 6 h and IL-6 at 24 h were independently associated with the effectiveness of the reperfusion therapy (OR: 1.004; 95% CI: 1.002–1.006; *p* < 0.0001 and OR: 0.979; 95% CI: 0.967–0.992; *p* = 0.001). Table 4 shows the measured levels of all the studied biomarkers.

Simultaneous analysis of these two values in the logistic regression model revealed a positive association for MMP-9 levels at 6 h with effective reperfusion (OR: 1.008; 95% CI: 1.005–1.010; *p* < 0.0001). In contrast, IL-6 at 24 h was an independent factor strongly associated with reperfusion failure (OR: 0.958; 95% CI: 0.942–0.973; *p* < 0.0001) (Table 5).

### 3.5. Association of Temperature with HT and Infarct Volume

In this study, 68% (*n* = 595) of patients did not have HT. Among the patients who developed HT, IH1 was the most common subtype (*n* = 187, 21.4%), followed by IH2 (*n* = 46, 5.3%), PH1 (*n* = 26, 3.0%), and PH2 (*n* = 21, 2.4%). In all groups, temperature was directly related to haemorrhagic transformation and higher temperatures were observed in the most severe cases. Patients with no HT or IH showed an increase in temperature from admission to 6 h that then declined at 24 h, while PH patients showed an increase in temperature over time. The multivariate analysis showed an association between PH and temperature (OR: 3.468; 95% CI: 1.368–8.792, *p* = 0.009), at 6 h (OR: 3.231; 95% CI: 1.254–8.326; *p* = 0.015) and at 24 h (OR: 4.588; 95% CI: 1.726–12.199, *p* = 0.002).

Regarding infarct size, the mean infarct volume at days 4–7 was 51.1 ± 75.8 mL. We observed no correlation between infarct volume and temperature at 6 h (*r* = 0.066; *p* = 0.066), although a significant correlation was observed at admission (*r* = 0.110; *p* = 0.001) and at 24 h (*r* = 0.302; *p* < 0.0001). Multivariate analysis showed no association between infarct volume and temperature at admission (B: 6.956; 95% CI: 0.620–14.531; *p* = 0.072) or at 6 h (B: 6.765; 95% CI: 0.725–14.253; *p* = 0.077). However, patients with higher temperatures at 24 h were over 20 times more likely to present greater infarct volumes between days 4 and 7 (B: 23.369; 95% CI: 16.308–30.430; *p* < 0.0001).

## 4. Discussion

Consistent with the findings of our previous study [2], higher body temperature at 24 h in the present study was negatively associated with effective reperfusion. This effect was also reflected by increased infarct volumes and poor outcome at 3 months in patients treated with rtPA. Higher body temperature at admission was also negatively associated with effective reperfusion but did not impair functional outcome at 3 months. Contrary to the observations at 24 h, our analysis showed that temperature at 6 h in rtPA patients was not associated with poor outcome or infarct volume. Higher body temperature at 6 h was positively associated with effective reperfusion, although this association disappeared after adjusting for confounding factors in the logistic regression analysis.

Although the positive association observed between mild temperature at 6 h and effective reperfusion was weak, previous studies support the beneficial effect of mild hyperthermia in rtPA-treated patients [16,32,33,34,35]. Indeed, the results of in vitro studies on rtPA activity are consistent with this rationale and demonstrated a direct relationship between high body temperature and clot lysis effectiveness [20]. In this regard, in the Paracetamol (Acetaminophen) In Stroke (PAIS) trial aimed to assess whether early treatment (12–24 h) with paracetamol improved functional outcome in patients with acute stroke by reducing body temperature and preventing fever, reporting that this analgesic compound did not improve functional outcomes [32]. A second sub-analysis, derived from the same PAIS trial and designed to evaluate the influence of baseline body temperature on the effect of rtPA (alteplase) and functional outcome in patients with acute IS, found that high body temperature might have a larger benefit for treatment with alteplase than that in patients with lower body temperature [33]. In brief, during the initial 6 h window after cerebral infarction when rtPA treatment was provided, the results of our analysis suggested that the effect of high body temperature on clot lysis was more relevant in terms of functional prognosis than the use of neuroprotective strategies related to hypothermia. Therefore, efforts are required to develop novel approaches to improve thrombolytic therapy [36].

Analysis of thrombolytic biomarkers showed that MMP-9 levels at 6 h were directly correlated with temperature and with the effectiveness of reperfusion therapy, which could be explained by the increase in rtPA activity, although with an increased risk of developing HT, as was observed in the analysis. These findings suggest that the threshold of the beneficial effect of body temperature is very narrow. Indeed, the beneficial effect of body temperature at 6 h was observed at an average temperature of 36.6 ± 0.7 °C.

As an inflammatory biomarker, IL-6 levels at 24 h were correlated with higher body temperature and negatively associated with effective reperfusion. High serum levels of inflammatory markers in the first 24 h were previously linked with early clinical deterioration and worsened outcome in acute IS [37,38,39]. These findings are further supported by our recent publication, in which IL-6 levels at 24 h were associated with worsened clinical outcomes in IS patients who underwent recanalisation therapy, regardless of the reperfusion effectiveness [40]. Therefore, delayed (24 h) episodes of fever or high temperature may amplify the ischaemic lesion mediated by an inflammatory response, overcoming the benefit of rtPA treatment.

Based on these clinical findings, exhaustive monitoring of mild hyperthermia in clearly defined ranges during rtPA administration (in the first 6 h) could have a beneficial effect on reperfusion therapies when combined with hypothermia strategies in later stages of the acute phase (i.e., at 24 h).

The present study has some limitations. This single-centre study was conducted on a retrospectively enrolled patient registry. Transcranial Doppler used to determine the reperfusion rate could only be performed in 18% of the treated patients. Therefore, an 8-point or more improvement in NIHSS in the first 24 h was chosen as the endpoint as it was sensitive to the effects of early reperfusion, consistent with the results of other analyses [41,42], although the use of this clinical criterion to define effective reperfusion may lead to a bias in the classification of therapeutic response groups. The lack of imaging analysis to correlate the location of occlusions with the reperfusion effectiveness is also a critical issue that could interfere with our analysis, as patients with distal occlusions usually have higher reperfusion rates, small infarct sizes, and possibly low temperatures on admission (low inflammatory response). To overcome this critical limitation, in this study, we defined effective reperfusion as a reduction of ≥ 8 points in the NIHSS in the first 24 h, which normally involves patients with more proximal occlusions. Finally, as none of the patients included in this study reached hyperthermic values, we cannot rule out the possibility that temperatures higher than 37.5 °C at 6 h could further promote effective reperfusion, although our data seems to predict such an effect.

In conclusion, the results of our analysis support the theory that mild hyperthermia could benefit reperfusion therapies by promoting clot lysis during the first 6 h of ischaemic stroke but worsen the outcome at later stages. Thrombolytic activity at 6 h seems to be related to temperature and may improve reperfusion therapies, warranting future studies to further elucidate the therapeutic potential of controlled mild hyperthermia in combination with rtPA administration.

## Figures and Tables

**Table 1:** Description of the 875 patients included in the study.

Characteristic	Total Sample (*n* = 875)
Age, years	72.0 ± 12.5
Women, %	45.9
Onset-treatment time, min	161.8 ± 61.2
Previous disability (modified Rankin Scale)	0 (0,0)
Hypertensive, %	63.8
Diabetics, %	22.9
Smokers, %	22.5
Alcohol consumption, %	10.3
Dyslipidaemia, %	39.3
Peripheral arterial disease, %	6.7
Atrial fibrillation, %	22.9
Ischemic heart disease, %	12.7
Temperature at admission, °C	36.2 ± 0.6
Temperature at 6 h, °C	36.6 ± 0.7
Temperature at 24 h, °C	36.5 ± 0.7
Blood glucose, mg/dL	138.6 ± 55.7
Glycosylated haemoglobin, %	6.2 ± 4.5
Leukocytes, × 10^3^/mL	8.3 ± 3.2
Fibrinogen, mg/dL	414.2 ± 103.2
Microalbuminuria, mg/24 h	5.7 ± 7.8
C-reactive protein, mg/L	5.5 ± 4.7
LDL-cholesterol, mg/dL	108.0 ± 40.7
HDL-cholesterol, mg/dL	41.7 ± 14.9
Triglycerides, mg/dL	114.2 ± 51.5
Sedimentation rate, mm	18.3 ± 20.4
NIHSS at admission	17 (12,22)
DWI volume at admission, mL	28.1 ± 43.5
Leukoaraiosis	
No, %	41.7
Fazekas I, %	36.5
Fazekas II, %	15.0
Fazekas III, %	6.9
Trial of ORG 10172 in acute stroke treatment (TOAST)	
Atherothrombotic, %	23.5
Cardioembolic, %	43.5
Lacunar, %	1.3
Undetermined/others, %	31.7
Type of reperfusion treatment	
Systemic fibrinolysis, %	91.1
IV or IA thrombolysis + thrombectomy, %	8.9
Effective reperfusion, %	44.0
Early neurological deterioration, %	9.9
Haemorrhagic transformation	
No, %	68.0
IH1, %	21.4
IH2, %	5.3
PH1, %	3.0
PH2, %	2.4
Lesion volume at days 4–7, mL	51.1 ± 75.8
Modified Rankin Scale at 3 months	1 (0,3)
Good functional outcome at 3 months, %	69.3

LDL: low density lipoprotein; HDL: high density lipoprotein; NIHSS: National Institute of Health Stroke Scale; DWI: diffusion-weighted imaging; IH1: infarction type 1; IH2: infarction type 2; PH1: parenchymal haemorrhage type 1; PH2: parenchymal haemorrhage type 1; IV: intravenous; IA: intra arterial.

**Table 2:** Logistic regression model for temperature at three different time points associated with effective reperfusion.

	OR *	CI 95%	*p*
Temperature at admission	0.700	0.552–0.887	0.003
Temperature at 6 h	1.178	0.934–1.484	0.166
Temperature at 24 h	0.373	0.290–0.480	0.001

* Adjusted for age, sex, onset-treatment time, blood glucose, atrial fibrillation, National Institute of Health Stroke Scale (NIHSS) score at admission, leukoaraiosis, and haemorrhagic transformation.

**Table 3:** Logistic regression model for temperature at three different time points showing the relationships to poor outcomes at 3 months.

	OR *	CI 95%	*p*
Temperature at admission	0.817	0.475–1.405	0.465
Temperature at 6 h	0.792	0.934–1.484	0.792
Temperature at 24 h	2.190	1.264–3.793	0.005

* Adjusted for age, sex, onset-treatment time, blood glucose, atrial fibrillation, National Institute of Health Stroke Scale (NIHSS) score at admission, diffusion-weighted imaging (DWI) volume at admission, leukoaraiosis, and effective reperfusion.

**Table 4:** Levers of biomarkers analysed at 6 and 24 h.

	*N*	Minimum	Maximum	Mean ± Standard Deviation
MMP-9 (6 h; ng/mL)	799	3.6	430.1	103.81 ± 79.16
Cellular fibronectin (6 h; ng/mL)	804	4.1	35.9	8.55 ± 4.55
TNF-α (Basal; ng/mL)	831	1.8	45.8	16.84 ± 6.65
IL-6 (Basal; ng/mL)	841	1.1	58.6	11.17 ± 10.00
MMP-9 (24 h; ng/mL)	653	3.6	339.1	87.66 ± 60.55
Cellular fibronectin (24 h; ng/mL)	662	1.7	18.3	7.11 ± 2.260
TNF-α (24 h; ng/mL)	669	7.3	65.8	27.98 ± 9.16
IL-6 (24 h; ng/mL)	666	8.4	83.6	32.45 ± 13.88

MMP-9: matrix metalloproteinase 9; TNF-α: tumour necrosis factor-alpha; IL-6: interleukin 6.

**Table 5:** Logistic regression model for the levels of biomarkers related to effective reperfusion.

	OR *	95%CI	*p*
MMP-9 levels at 6 h (ng/mL)	1.008	1.005–1.010	<0.0001
IL-6 levels at 24 h (pg/mL)	0.958	0.942–0.973	<0.0001

* Adjusted for age, sex, onset-treatment time, blood glucose, atrial fibrillation, National Institute of Health Stroke Scale (NIHSS) score at admission, and leukoaraiosis. MMP-9: matrix metalloproteinase 9; IL-6: interleukin 6.
